# Divergent Roles of *Salmonella* Pathogenicity Island 2 and Metabolic Traits during Interaction of *S. enterica* Serovar Typhimurium with Host Cells

**DOI:** 10.1371/journal.pone.0033220

**Published:** 2012-03-12

**Authors:** Stefanie U. Hölzer, Michael Hensel

**Affiliations:** 1 Mikrobiologisches Institut, Universitätsklinikum Erlangen, Erlangen, Germany; 2 Abteilung Mikrobiologie, Universität Osnabrück, Osnabrück, Germany; Indian Institute of Science, India

## Abstract

The molecular mechanisms of virulence of the gastrointestinal pathogen *Salmonella enterica* are commonly studied using cell culture models of infection. In this work, we performed a direct comparison of the interaction of *S. enterica* serovar Typhimurium (*S.* Typhimurium) with the non-polarized epithelial cell line HeLa, the polarized cell lines CaCo2, T84 and MDCK, and macrophage-like RAW264.7 cells. The ability of *S.* Typhimurium wild-type and previously characterized auxotrophic mutant strains to enter host cells, survive and proliferate within mammalian cells and deploy the *Salmonella* Pathogenicity Island 2-encoded type III secretion system (SPI2-T3SS) was quantified. We found that the entry of *S.* Typhimurium into polarized cells was much more efficient than entry into non-polarized cells or phagocytic uptake. While SPI2-T3SS dependent intracellular proliferation was observed in HeLa and RAW cells, the intracellular replication in polarized cells was highly restricted and not affected by defective SPI2-T3SS. The contribution of aromatic amino acid metabolism and purine biosynthesis to intracellular proliferation was distinct in the various cell lines investigated. These observations indicate that the virulence phenotypes of *S.* Typhimurium are significantly affected by the cell culture model applied.

## Introduction

*Salmonella enterica* is a common Gram-negative pathogen of man and animals. These invasive, facultative intracellular bacteria can infect a variety of host organisms [1]. Within the host, *S. enterica* can adopt an intracellular lifestyle in epithelial cells, fibroblasts, macrophages, dendritic cells and other immune cells. Virulence factors of *S. enterica* serovar Typhimurium (*S.* Typhimurium) can be studied in small animal models, such as mouse models for systemic pathogenesis as well as for intestinal inflammation. In addition, various *in vitro* models that deploy primary cells or immortalized cell lines have been proven useful for the study of molecular mechanisms during host-pathogen interaction in *S. enterica* infections.

In addition to the uptake by phagocytic cells, *S.* Typhimurium actively invades non-phagocytic cells by means of the *Salmonella* Pathogenicity Island 1 (SPI1)-encoded type III secretion system (T3SS). The effector proteins translocated by the SPI1-T3SS remodel the host cell actin cytoskeleton resulting in internalization of the bacteria [2]. Intracellular survival and replication mainly depends on the function of a second T3SS encoded by SPI2 [3]. Effector proteins of the SPI2-T3SS result in a remodeling of the endosomal system and the maturation of the pathogen-containing compartment in host cells [4]. Intracellular *S.* Typhimurium reside within a specialized membrane-compartment termed *Salmonella*-containing vacuole or SCV. Depending on the nature of the infected host cell, *S.* Typhimurium persist or proliferate within the SCV. The SCV is considered as a nutrient restricted and hostile environment that imposes a series of stress stimuli on intracellular bacteria [4]. One prominent consequence of the intracellular activities of *S.* Typhimurium is the induction of extensive networks of tubular endosomal membrane compartments, among these *Salmonella*-induced filaments or SIFs have been studied in most detail [5].

Intracellular survival and replication of *S.* Typhimurium also depends on the metabolic flexibility and adaptation to the intracellular environment. Earlier work has demonstrated that mutant strains of *S.* Typhimurium that are auxotrophic for distinct amino acids or precursors of nucleotide biosynthesis [6], [7] are defective in intracellular proliferation. These mutants strains are highly attenuated in virulence and some of the strains have proven useful as attenuated life vaccine against *S. enterica* infections or live carrier strains for heterologous vaccines [6].

Mammalian cell lines commonly used to study the intracellular phenotypes of *S.* Typhimurium include murine macrophage-like cells such as cell line RAW264.7 and the human epithelial cell line HeLa. These cells are adherent epithelial cells that form monolayers, however HeLa cells do not differentiate into a polarized epithelial layer-like organization. We have recently reported that cell invasion, a hallmark virulence function of *S.* Typhimurium, is distinct in infection models with non-polarized and polarized epithelial cells [8]. For example, invasion of polarized epithelial cells requires the coordinated function of the SPI1-encoded T3SS and the SPI4-encoded T1SS [8]. Previous studies also demonstrated that intracellular phenotypes of *S.* Typhimurium in fibroblasts are distinct from macrophages and epithelial cells (reviewed in [9]). Furthermore, the intracellular growth rate *in vivo* appears much lower than the intracellular replication observed in most cell culture models (reviewed in [10]).

These various observations prompted us to perform a comparative investigation of the characteristics of *S.* Typhimurium in various commonly used cell culture models. In addition to RAW and HeLa cells, the canine kidney epithelial cell line MDCK, and the human colonic cell lines CaCo2 and T84 were used. MDCK, CaCo2 and T84 can be differentiated to form polarized epithelial layers. We observed striking cell line-specific differences of *S.* Typhimurium (i) in the ability to enter non-phagocytic cells, (ii) to proliferate within host cells, as well as in the roles (iii) of metabolic pathways, and (iv) of the SPI2-T3SS for the adaptation to the intracellular lifestyle.

## Materials and Methods

### Bacterial strains and growth conditions

*Salmonella enterica* serovar Typhimurium strain NCTC 12023 (*S.* Typhimurium) was used as wild-type (WT) strain in this study and mutant strains were isogenic to this strain. P2D6 is deficient in *ssaV*, a structural component of the SPI2-encoded T3SS. Strain MvP473 is deleted in *aroA* encoding 3-phosphoshikimate 1-carboxyvinyltransferase required for synthesis of aromatic amino acids as well as for the synthesis of para-aminobenzoic acid (pABA) and para-hydroxybenzoic acid (DHBA). The *purD*-deleted strain MvP481 is deficient in ribosylamineS-phosphate:glycine-ligase (ADP) which is required for de novo synthesis of purine nucleotides. The mutations of strains P2D6, MvP473 and MvP481 were transferred into the *invC*-deficient strain MvP818 using P22 transduction according to standard methods [11]. The characteristics of strains and plasmids used in this study are listed in Table 1.

**Table 1:** Bacterial strains and plasmids used in this study.

Designation	genotype	relevant characteristics	reference
*Salmonella enterica* serovar Typhimurium strains			
NCTC 12023		wild type	National Collection of Type Cultures, Colindale, UK
P2D6	*ssaV*::mTn*5*	SPI2-T3SS defect, Kan^R^	[12]
MvP473	*ΔaroA*::*aph*	auxotrophic, Kan^R^	[13]
MvP481	Δ*purD*::*aph*	auxotrophic, Kan^R^	[8]
MvP818	Δ*invC*::FRT	SPI1-T3SS defect	[8]
MvP1436	Δ*invC*::FRT *ssaV*::mTn*5*	SPI1-T3SS, SPI2-T3SS defect	this study
MvP1437	Δ*invC*::FRT *purD*::*aph*	SPI1-T3SS defect, auxotrophic	this study
MvP1438	Δ*invC*::FRT *aroA*::*aph*	SPI1-T3SS defect, auxotrophic	this study
Plasmids			
pFPV25.1		const. eGFP	[35]
p2777		const. eGFP, *sseJ*::HA, low copy number vector	[21]

Bacterial strains were routinely grown in LB broth or on LB agar with 50 µg×ml^−1^ kanamycin or carbenicillin if required for the selection of markers or plasmids. Growth in minimal medium PCN containing 1 mM inorganic phosphate at pH 7.4 was performed as described before [14]. If indicated, media were supplemented with 40 µg×ml^−1^ each of L-tyrosine, L-tryptophan and L-phenylalanine and/or 10 µg×ml^−1^ each of 2,3-dihydrobenzoic acid and para-aminobenzoic acid (all from Sigma, Taufkirchen) for the *aroA*-deficient strain, and 1 mM adenine hemi-sulfate (Sigma) for the *purD*-deficient strain. Alternatively, amino acids were replaced by 0.1% casamino acids (Difco, BD, Heidelberg).

### Cell culture

Various mammalian cell lines were used in this study. HeLa is a human epithelial cancer cell line with adherent, non-polarized growth (obtained from Cell Lines Service CLS, Heidelberg,). The C2BBE1 clone of CaCo2 (CaCo2) (American Type Culture Collection, ATCC) and T84 (European Collection of Cell Cultures, ECACC) are human colonic epithelial cells lines that form polarized monolayers. MDCK is a canine kidney epithelial cell line that also forms polarized monolayers (MDCK subline pf, obtained from Prof. Dr. M. Goppelt-Struebe, Med. Klinik 4, Universitätsklinikum Erlangen) and cultured as described before [15]. The polarization of CaCo2 and T84 cells, but not of MDCK cells can be controlled by determination of the transepithelial electrical resistance (TEER). RAW264.7 is a murine macrophage-line cell line with the ability to phagocytose particulate materials (CLS). Cell line cultures were routinely maintained in DMEM (PAA) in an incubator at 37°C with a humidified atmosphere containing 5% CO_2_. For cultivation for prolonged periods of time, for example to induce the formation of polarized monolayers, a mixture of penicillin/streptomycin was added.

For microscopy, cells were seeded on glass cover slips placed in the wells of 24-well cell culture plates. For experiments with polarized cell lines, cells were grown directly in wells of 24 well plates. Alternatively, CaCo2 and T84 cells were grown in the upper reservoir of transwell membrane filter inserts with a pose size of 0.45 µm (transwell, Millipore) in order to allow the development of a polarized monolayer. DMEM medium with 4.7 g×l^−1^ glucose, 2 mM L-glutamine, 10 µg×ml^−1^ holo-transferin and lacking Na-pyruvate was used for these cultures.

For CaCo2 and T84 cell grown in transwell filter inserts, the TEER was determined over the course of the culture. The TEER for CaCo2 monolayers increased to 600–800 Ω and maintained at this level for several days. In this state, the cells were considered as polarized. For T84 monolayers, culture on transwell inserts was performed until the TEER increased to about 2,000 Ω and maintained at this level.

### Invasion and intracellular replication assays

The internalization of *S.* Typhimurium strains by various cell lines was determined by gentamicin protection assays. For infection of RAW264.7 cells, stationary cells from overnight cultures were used. Infection of RAW264.7 cells with SPI1-deficient strains and all other infection experiments with epithelial cells were performed with cultures diluted 1∶31 in LB broth from overnight cultures and sub-cultured for 3.5 h in glass test tubes with agitation in a roller drum. These culture conditions induced the expression of invasion genes. The bacterial inoculum was applied at various multiplicities of infection as specified for the respective experiment. After infection of 30 min, non-internalized bacteria were removed by washing with prewarmed PBS and remaining extracellular bacteria were killed by incubation in cell culture media containing 100 µg×ml^−1^ gentamicin for 1 h. For invasion assays, cells were washed and lysed. For intracellular replication assays, the medium was changed to medium containing 10 µg×ml^−1^ gentamicin until lysis of the infected cells at the indicated time points after infection. In order to determine the number of viable intracellular *S.* Typhimurium, host cells were washed with prewarmed PBS and lysed by the addition of 0.1% Triton X-100 in PBS for HeLa, RAW cells, or 0.5% desoxycholate in PBS for MDCK, T84, or CaCo2 cells. Serial dilutions of the lysates were plated onto Müller-Hinton Agar for the quantification of colony-forming units. Rates of internalization were calculated as percentage of the CFU of the inoculum recovered as gentamicin-protected bacteria from host cells. Rates of intracellular proliferation were calculated as ratio of gentamicin-protected bacteria recovered from host cells at the time points compared.

### Immunofluorescence microscopy

Culture of cells and infection was performed as described above. The various *S.* Typhimurium strains harbored pFPV25.1 for the constitutive expression of GFP for visualization of intracellular bacteria. Cells were fixed by addition of 3% para-formaldehyde in PBS for 20 min, followed by permeabilization with 0.1% saponin in PBS containing BSA and goat serum. For detection of host cell endosomal membranes, immuno-staining for LAMP-2 was performed. The analyses of SPI2-T3SS-dependent translocation was performed by detection of effector protein SseJ tagged with the HA epitope. Translocated SseJ-HA was detected by immuno-staining with rat monoclonal antibody against HA (Roche). Primary antibody binding was detected by Alexa-conjugated secondary antibodies (Jackson, Dianova, Hamburg, Germany). The host cell actin cytoskeleton was labeled using an Alexa–Phalloidin conjugate (Molecular Probes, Heidelberg, Germany). Epi-fluorescence imaging was performed using a Zeiss Aviovert 200 M wide-field microscope. The Axiovision 4.8 software was used for image acquisition and analyses and subsequently micrographs were assembled using Adobe PhotoShop CS4.0.

### Data analyses

Statistical analysis was performed by one-way ANOVA with Boneferri test using SigmaPlot 11.0. Statistical significance is indicated as n.s. not significant; *, P<0.05; **, P<0.01; ***, P<0.001.

## Results

### Internalization of *S.* Typhimurium by various host cell lines

**Figure 1:**
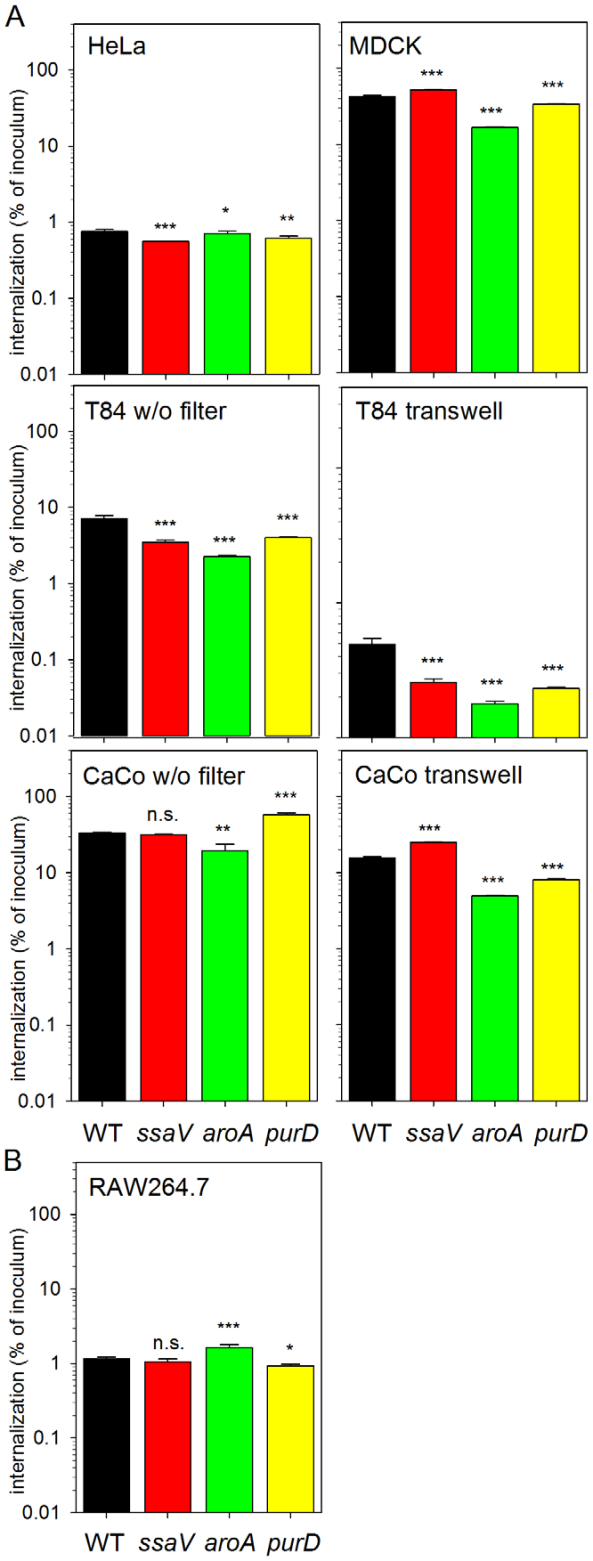
Internalization of *S.* Typhimurium by various cell lines. A) Invasive *S.* Typhimurium WT (black bars), and isogenic SPI2-deficient (*ssaV*, red bars) or auxotrophic Δ*purD* (green bars) and Δ*aroA* (yellow bars) strains were used to infect various host cell lines at an MOI of 1. HeLa and MDCK epithelial cells were grown in multiwell dishes. The human epithelial cell lines T84 and CaCo2 were either grown directly in multiwell dishes (w/o filter) or using transwell membrane filter inserts in order to allow differentiation to polarized monolayers (transwell). After invasion for 1 h and killing non-internalized bacteria by addition of gentamicin, the cells were lysed by addition of 0.1% Triton X-100 in PBS (HeLa, RAW) or 0.5% desoxycholate in PBS (MDCK, T84, CaCo2), and serial dilutions of lysates were plated onto agar plates for determination of the amount of internalized bacteria. The internalization is expressed as percentage of the bacterial inoculum applied for invasion. B) Internalization of *S.* Typhimurium by the murine macrophage-like cell line RAW264.7 was quantified as for A), except that bacteria from stationary cultures were used for infection at an MOI of 1. The graphs show means and standard deviations of assays performed in triplicate and data sets are representative of three independent experiments are shown. Statistical significances were calculated for mutant vs. WT strains and indicated as n.s., not significant; *, P<0.05; **, P<0.01; ***, P<0.001.

For the characterization of the interaction of *S.* Typhimurium with various host cell lines, invasion by wild-type (WT) *S.* Typhimurium, a SPI2-deficient strain (*ssaV*) and *aroA*- or *purD*-deficient mutant strains was analyzed (Fig. 1). The bacteria were sub-cultured to late log growth phase in medial broth in order to induce the expression of genes required for invasion. For the infection assays, T84 and CaCo2 cells are either grown directly on surfaces of cell culture dishes or on transwell filter systems. Under our laboratory conditions, MDCK cells formed polarized monolayers if cells were grown for 3–5 days on conventional cell culture dishes. For T84 and CaCo2 cells, the differentiation into polarized monolayers can be induced by the growth on filter inserts with defined pore size (transwell system). The formation of polarized monolayers was judged by the expression of the apical brush border and continuous tight junctions (MDCK), or by increase of the transepithelial resistance for T84 and CaCo2 cells grown in transwell filters.

Invasion of non-polarized HeLa cells was similar for WT, *ssaV*, *purD*, and *aroA* strains and 0.5–0.8% of the inoculum was internalized (Fig. 1A). Much higher rates of entry were observed for MDCK cells and up to 42% of the WT inoculum applied was Gentamicin-protected and could be recovered from lysed MDCK cells. The entry of the *aroA* strain was reduced to 17%. The quantification of entry into T84 cells indicated approximately similar internalization, especially if T84 cells grown on transwell filters were used. Internalization into CaCo2 cells was in a similar range for all strains (Fig. 1A).

To test if the internalization of the various mutant strains used in this study is affected by defective SPI2-T3SS or auxotrophies, we quantified the passive uptake by phagocytosis in RAW264.7 cells. Using stationary, non-invasive *S.* Typhimurium strains, about 1% of the bacterial inoculum was internalized by RAW264.7 and only minor variations were observed for the different strains tested (Fig. 1B).

These results demonstrate that the invasion of polarized cells by *S.* Typhimurium occurs with much higher efficiency than invasion of non-polarized cells. Remarkable is the more than 50-fold higher number of *S.* Typhimurium internalized by MDCK cells compared to HeLa cells as non-polarized epithelial cells. Uptake by RAW cells was neither affected by the defect of the SPI2-T3SS, nor *aroA* or *purD* mutations. In contrast, the invasion of polarized cells by the *aroA* strain was reduced.

### Intracellular proliferation of *S.* Typhimurium in various host cell lines

**Figure 2:**
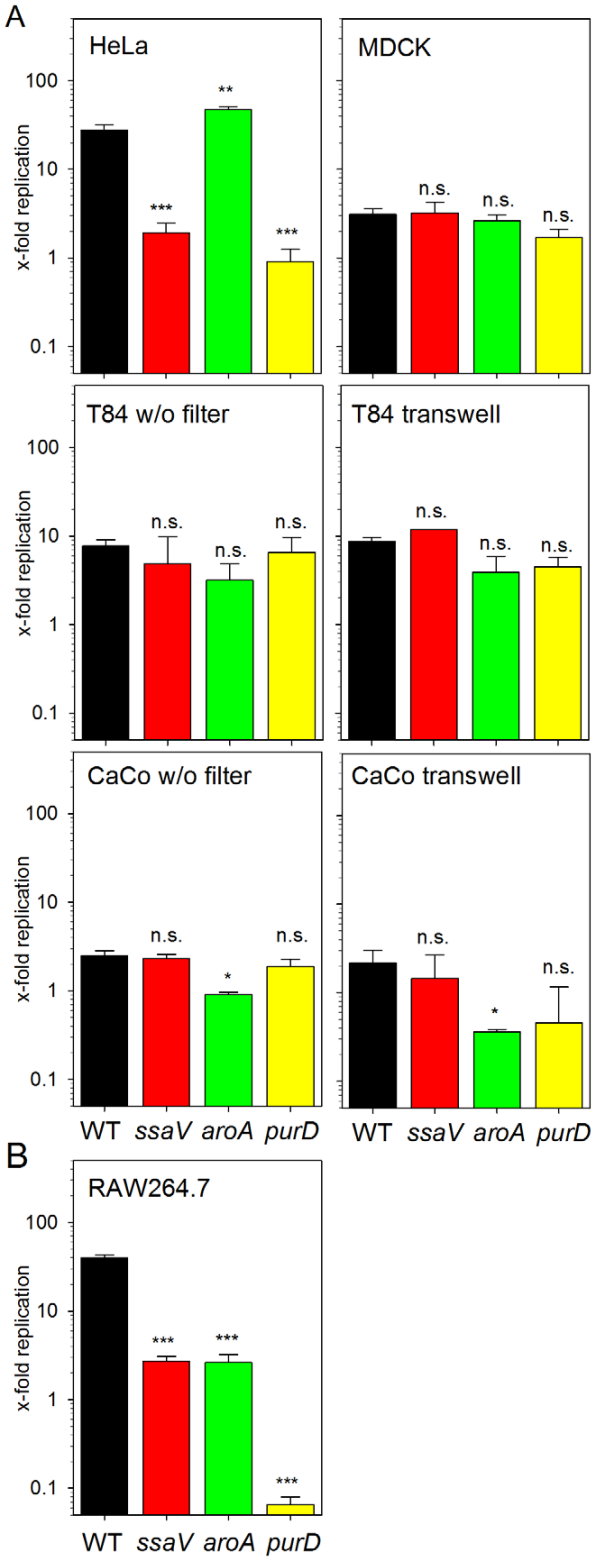
Intracellular proliferation of *S.* Typhimurium in various cell lines. *S.* Typhimurium strains were used for infection of epithelial cell lines (A) or macrophage-like RAW 264.7 cells (B) at an MOI of 1 as described for Fig. 1. Non-internalized bacteria were killed by the addition of gentamicin and infected host cells were lysed at 2 h or 16 h after infection in order to determine the number of intracellular bacteria. The x-fold intracellular replication is the ratio of intracellular bacteria at 16 h divided by the 2 h values. The graphs show means and standard deviations of assays performed in triplicate and data sets representative of three independent experiments are shown.

We next quantified the intracellular replication in various eukaryotic cell lines. In RAW macrophages, *S.* Typhimurium WT showed about 40-fold replication over 14 h of intracellular presence (Fig. 2B). In line with previous studies [16], [17], the SPI2-deficient strain was highly reduced in intracellular replication with about 3-fold increase in intracellular bacteria over 14 h. The replication of the *aroA* strain was reduced to a similar rate, while the *purD* strain was more severely attenuated and only about 10% of the internalized bacteria were recovered after 14 h of intracellular presence. This observation indicated that the *purD* strain is more susceptible to killing mechanisms in macrophages.

Much more heterogeneous intracellular phenotypes of the strains were observed in epithelial cells. In HeLa cells, the *ssaV* and *purD* strains showed low replication rates of 1.9 and 0.9-fold, respectively. Intracellular replication of the *aroA* strain was similar than that of the WT (27.6- and 47.1-fold proliferation for WT and *aroA* strains, respectively). In MDCK cells, intracellular replication of all strains tested was highly reduced to about 2- to 3-fold increase of intracellular bacteria over 14 h and similar levels of replication were observed for CaCo2 cells. Interestingly, no significant reduction of intracellular survival and replication was observed for the *ssaV* strain in any of the polarized cell models. Intracellular replication of *S.* Typhimurium WT in T84 or CaCo2 cells was in the range of 8-fold and independent from the cultivation of cells on plastic surfaces or transwell filters. The auxotrophic strains showed rather small differences in intracellular replication in the various polarized cell models.

We found a remarkable difference in intracellular phenotypes of the *aroA*-deficient mutant in HeLa cells versus macrophages. While epithelial cells were invaded by the SPI1-mediated macropinocytosis, the infection of macrophages was mediated by phagocytosis of non-invasive bacteria. The different infection procedures are required, since SPI1-mediated invasion of phagocytic cells induces a rapid form of apoptotic cell death that interferes with the quantification of intracellular replication [18].

**Figure 3:**
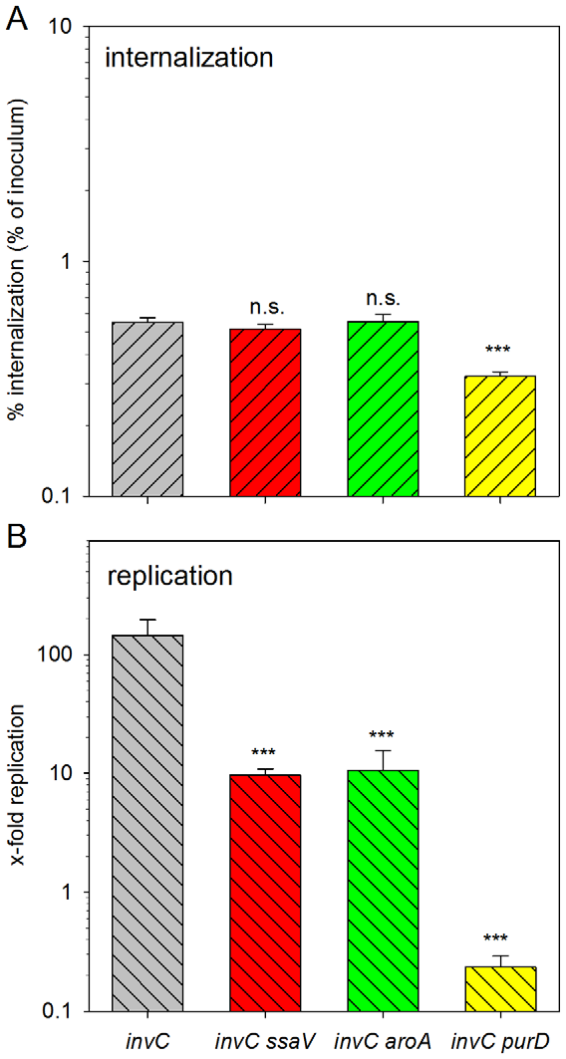
Effect of the bacterial growth phase on internalization and intracellular proliferation of various strains. *S.* Typhimurium strains deficient in *invC* and various other functions as indicated were used for infection of RAW264.7 macrophages at an MOI of 1. The bacteria were grown to late log phase prior to infection. The internalization (A) and intracellular proliferation (B) was determined as described for Fig. 1 and Fig. 2, respectively. Statistical significances were calculated for double mutant vs. *invC* strains and indicated as for **Fig. 1**.

Since invasive and non-invasive bacteria were in a different growth phase, we considered that metabolic status of the bacterial inoculum as one reason for the different replication phenotypes in HeLa and RAW cells. To test this hypothesis, we performed infection of macrophages with bacteria subcultured to the same growth phase as for invasion of epithelial cells. Since expression of the SPI1-T3SS and translocation of the SPI1-effector SipC is triggering host cell pyroptosis in phagocytic cells [19], the infection experiments were performed in an *invC* strain background that is non-invasive and unable to translocate SPI1 effector proteins. Under these conditions, the internalization was comparable between the strains tested (Fig. 3A). The intracellular replication of the *invC* strain was higher than that of the stationary phase WT strain and the *invC ssaV* or *invC aroA* strains showed about 10-fold reduced rates of proliferation (Fig. 3B). The *invC purD* strain showed a reduced intracellular survival similar to the results obtained for infections with stationary *purD* bacteria. From these observations we conclude that the growth state of the bacteria affects the dynamics of intracellular replication, but does not affect intracellular defects caused by auxotrophies.

**Figure 4:**
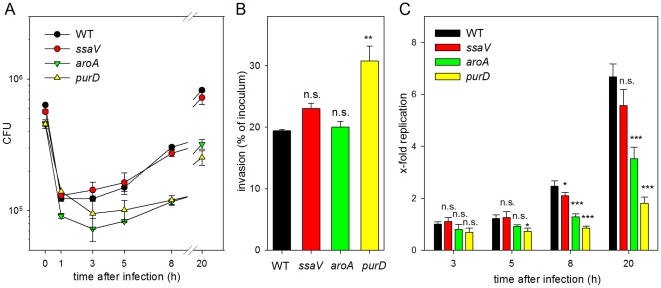
Kinetics of invasion and intracellular proliferation of *S.* Typhimurium in MDCK cells. Polarized monolayers of MDCK cells were infected with various *S.* Typhimurium strains with an MOI of 1 as described for **Fig. 1**. A) The amount of bacteria in the inoculum (0 h) was determined by plating of serial dilutions of the inoculum onto agar plates. The amount of intracellular bacteria was determined at various time points after infection as described for **Fig. 2**. B) The proportion of internalized bacteria was determined as described for **Fig. 1**. C) The x-fold intracellular replication is the ratio of intracellular bacteria at 3, 5, 8 or 20 h divided by the 1 h values. The graphs show means and standard deviations of assays performed in triplicates.

We next followed the kinetics of internalization and intracellular proliferation of *S.* Typhimurium in MDCK cells (**Fig. 4**). The amounts of viable bacteria were determined in the inoculum and for intracellular bacteria at various time points after infection (**Fig. 4**A) and the percentage of internalized bacteria was determined (**Fig. 4**B). Internalization of WT, *ssaV* and *aroA* strains was in a similar range, while higher uptake of the *purD* strain was observed. Similar to the results show in **Fig. 2**, the intracellular proliferation was rather low (**Fig. 4**C). Proliferation was observed for WT and *ssaV* strains from 8 h after infection, reaching a maximal rate of 6–fold proliferation at 20 h vs. 1 h after infection. Intracellular proliferation of the *aroA* strain was delayed and lower compared to the WT strain. Despite the higher rate of internalization, the intracellular proliferation of the *purD* strain was very low and an about 2-fold increase of intracellular CFU was observed during 19 h of intracellular presence.

In order to test if the reduced intracellular proliferation of the auxotrophic strains is a function of the reduced availability of growth factors in the culture medium, we determined the growth in bacteriological and cell culture media (**Table 2**). All strains grew to similar cell densities in LB broth after incubation for 24. In synthetic media (PCN minimal media), the WT and *ssaV*-deficient strains reached cell densities OD_600_ 4.2 and 4.1, respectively, while *purD*- or *aroA*-deficient strains showed no detectable growth. Growth of the *purD* strain in PCN minimal medium was fully restored by addition of 1 mM adenine hemi-sulfate (data not shown). Supplementation of PCN medium with 10 µg×ml^−1^ each para-aminobenzoic acid (pABA) and para-hydroxybenzic acid (DHBA) and 40 µg×ml^−1^ each of tyrosine, tryptophan and phenylalanine restored growth of the *aroA* strain to an final OD_600_ of 0.93. Supplementation of PCN with the same amounts of pABA and DHBA and addition of 0.1% casamino acids fully restored growth of the *aroA*-deficient strain to cell densities of the WT strain. These growth characteristics in bacteriological media were in line with previous observations for *purD*- and *aroA*-deficient strains [6], [20].

**Table 2:** Cell densities of *S.* Typhimurium WT and the *aroA*-deficient strain after 24 h growth in various media.

Medium	Supplements *	WT	*aroA*
LB	-	5.2**	5.3
PCN	-	4.2	0.08
PCN	pABA DHBA	4.55	0.12
PCN	F W Y	3.92	0.29
PCN	pABA DHBA F W Y	4.04	0.93
PCN	Casa	3.85	1.5
PCN	pABA DHBA Casa	5.25	4.75

* Supplements: pABA, DHBA, each at 10 µg×ml^1^, F, phenylalanine, W, tryptophan, Y, tyrosine, each at 40 µg×ml^1^, Casa, 0.1% casamino acids.

** The OD_600_ was recorded after 24 h growth of 3 ml cultures in test tubes with aeration by rotation in a roller drum at 37°C.

**Figure 5:**
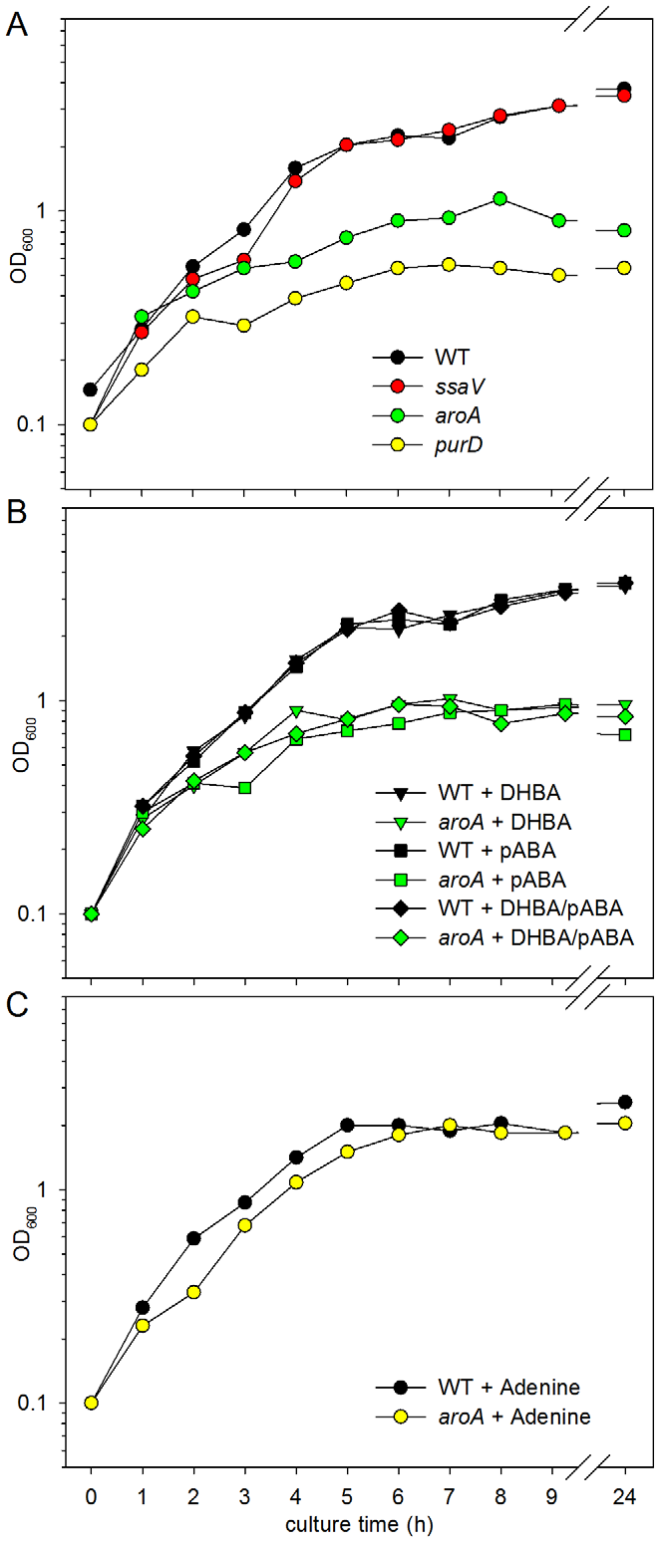
Growth of *S.* Typhimurium strains in cell culture media. A) *Salmonella* WT and various mutant strains were grown in DMEM cell culture medium without further additives. Cultures were grown in baffled flasks at 37°C with agitation at 180 rpm. Growth was recorded by determination of the OD_600_. B) Effect of supplementation of cell culture media on growth of the *aroA*-deficient strain. *S.* Typhimurium WT (black symbols) and the *aroA* strain (green symbols) were grown in DMEM supplemented with 10 µg×ml^−1^ 2,3- DHBA (triangles), 10 µg×ml^−1^ pABA (squares) or both supplements (diamonds). C) Effect of supplementation of DMEM with 1 mM adenine on growth of WT and *purD*-deficient strains. Growth curves are representative for three independent replicates with similar growth characteristics.

We next determined growth curves in DMEM cell culture medium (Fig. 5A). The growth of the *ssaV*-negative strain was comparable to the WT strains. In contrast, the growth of *aroA*- or *purD*-deficient strains rapidly slowed down. The final OD_600_ after 9 h of culture was 3.12 for the WT and *ssaV* strains, and 0.9 and 0.5 for *aroA*- and *purD*-deficient strains, respectively. This indicates that the auxotrophic strains lack important growth factors in DMEM that are present in rich media. To test if the growth defect of the *aroA* strain could be completed by addition of supplements to DMEM culture medium, pABA or DHBA or both compounds were added and growth was recorded (Fig. 5B). These supplements resulted in minute increase of cell densities during cultural growth, but did not fully restore growth to the level of the WT strain. This observation indicates that availability of aromatic amino acids restricts growth of the *aroA* strain in DMEM. By contrast, supplementation of the DMEM with adenine fully restored growth of the *purD*-deficient strain (**Fig. 5**C).

### Effect of auxotrophies on the function of the SPI2-T3SS and intracellular phenotypes

**Figure 6:**
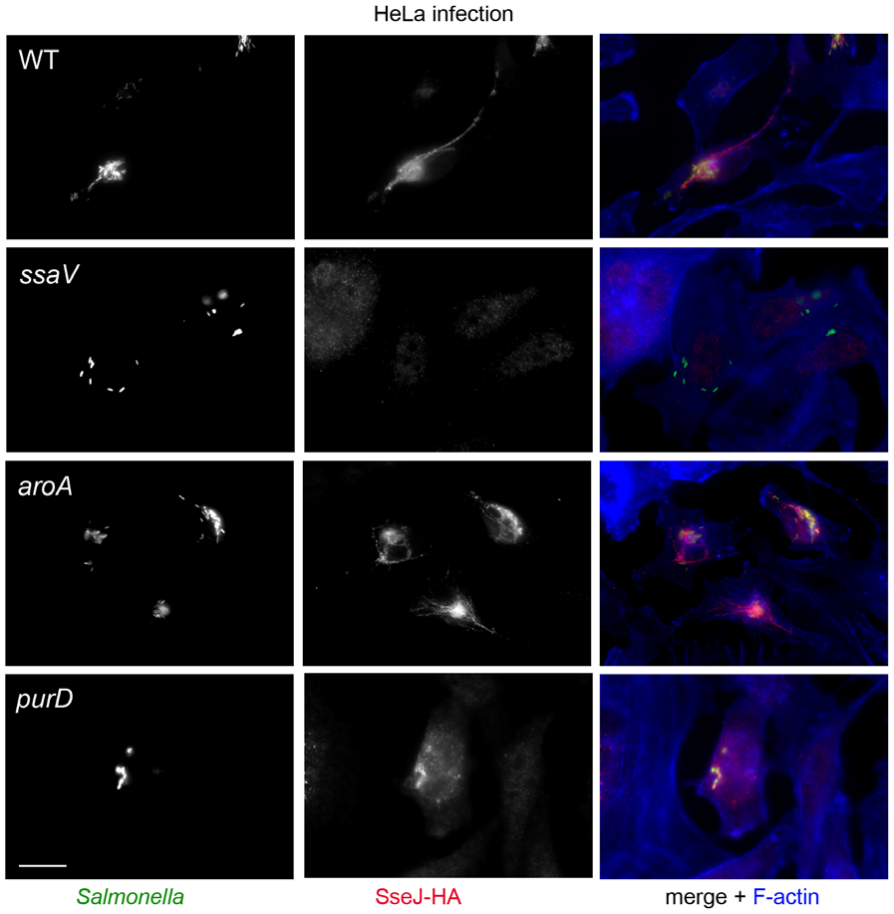
Effect of auxotrophic mutations on the function of the SPI2-encoded T3SS in HeLa cells. HeLa cells were infected with *S.* Typhimurium WT or various mutant strains each harboring a plasmid for the expression of *sseJ*::HA at an MOI of 10. At 16 h after infection, the cells were fixed and the translocated SseJ-HA was detected by immuno-staining with monoclonal antibody against HA (red). Intracellular bacteria were detected by virtue of GFP expression (green) and host cell actin was labeled with phalloidin-Alexa647 (blue). Representative cells for each infecting strain are shown. Scale bars represent 10 µm.

We next investigated the effect of mutations causing auxotrophies on the function of the SPI2-T3SS as a virulence factor with central importance for the intracellular lifestyle of *S.* Typhimurium in mammalian host cells. The translocation of SPI2-T3SS effector proteins by intracellular *S.* Typhimurium was determined using the SseJ-HA fusion protein as a representative marker (Fig. 6). As anticipated from our previous studies [21], [22], this effector was detected by immunofluorescence in an endosomal membrane-associated form after translocation by the WT strain, but no signals for SseJ-HA were obtained with the *ssaV* strain expressing *sseJ*::HA. Translocation of SseJ-HA was observed for the *aroA* and *purD* strains. There was no obvious difference in the apparent amount of SseJ-HA translocated by the different strains, but the signal intensity was dependent on the number of intracellular bacteria in an individual infected cell.

**Figure 7:**
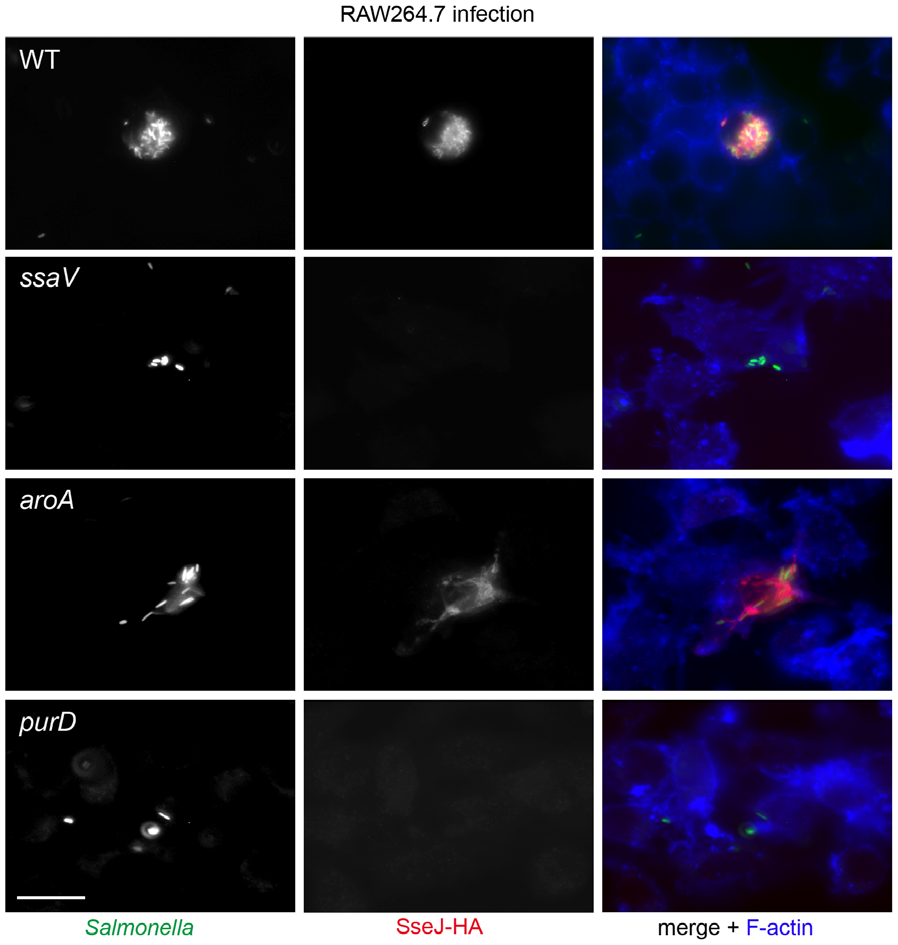
Effect of auxotrophic mutations on the function of the SPI2-encoded T3SS in RAW264.7 cells. Murine macrophage-like cells were infected with *S.* Typhimurium WT or various mutant strains each harboring a plasmid for the expression of *sseJ*::HA at an MOI of 10. Detection of translocated SseJ-HA was detected as described for Fig. 6. Representative cells for each infecting strain are shown. Scale bars represent 10 µm.

A different observation was made for translocation of SseJ-HA by the various strains in RAW264.7 cells (Fig. 7). The WT, but not the *ssaV* strain, translocated the effector protein into RAW cells. Signals for translocated SseJ-HA were also detected in macrophages infected with the *aroA* strain, but not in cells harboring the *purD* strain. It should be noted that the number of intracellular bacteria of the *purD* strain was highly reduced in macrophages, but also individual cells with a higher bacterial load were lacking signals for SseJ-HA.

**Figure 8:**
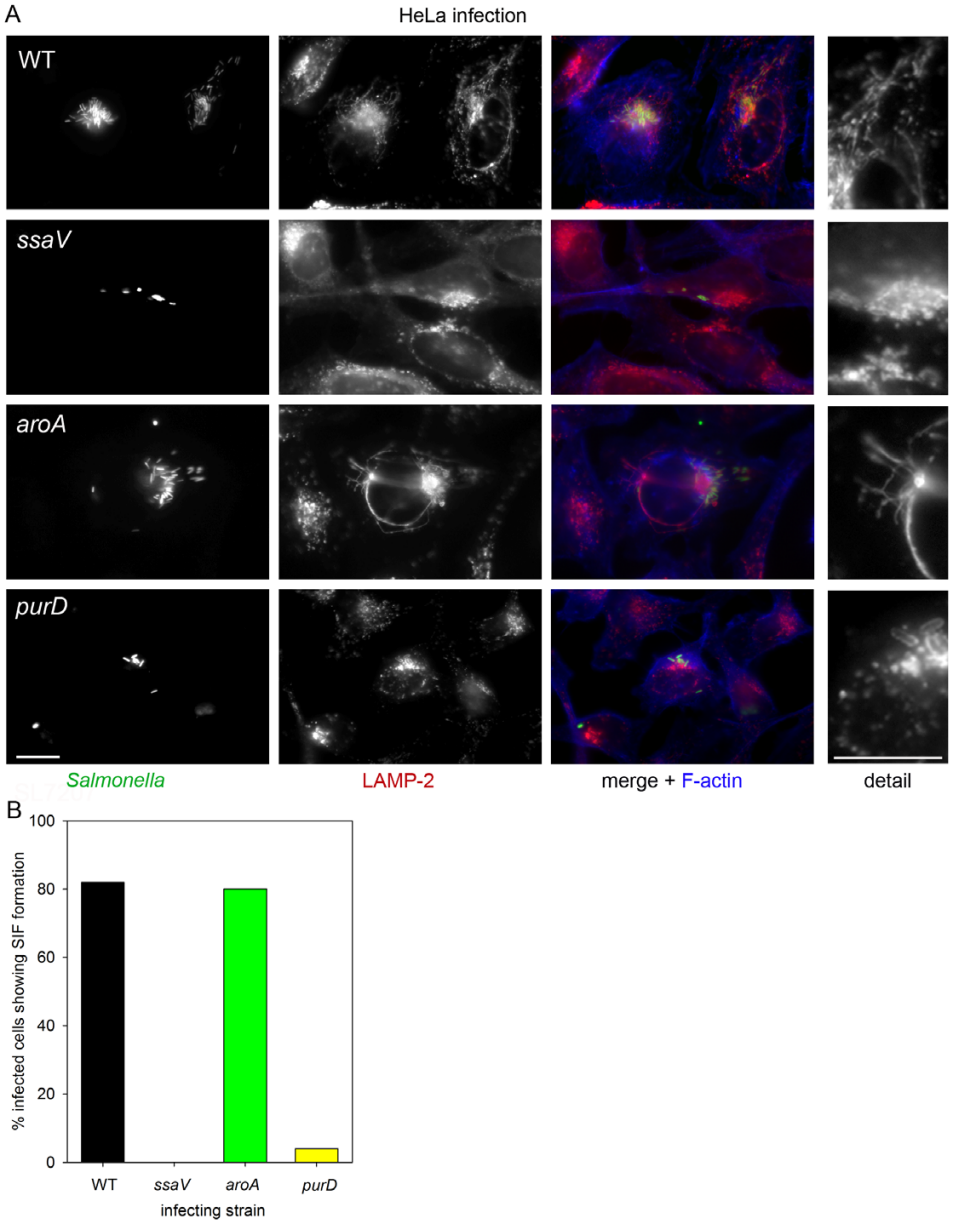
Effect of auxotrophic mutations on *S.* Typhimurium-induced phenotypes in HeLa cells. A) HeLa cells were infected with various *S.* Typhimurium strains as indicated at an MOI of 10. Cells were fixed 16 h after infection and immuno-stained for the host cell endosomal marker LAMP-2 (red) and F-actin was labeled with phalloidin-Alexa647. Note the appearance of tubular aggregates of LAMP-2-positive membranes or SIF in cells infected with WT and *aroA* strains. Scale bars represent 10 µm. B) Quantification of SIF formation in HeLa cells infected with various *S.* Typhimurium strains. For each strain, at least 50 infected cells were identified and examined for SIF formation. The percentage of SIF-positive cells is shown for one representative experiment of three repetitions.

Finally, we investigated the modification of the host cell by effector functions of intracellular *S.* Typhimurium. One prominent phenotype is the formation of SIF, resulting from the extensive aggregation of late endosomal/lysosomal vesicles of the host cell. SIF formation is dependent on the function of the SPI2-encoded T3SS and a subset of effector proteins. SIF formation was observed in HeLa cells infected with the WT strain and absent in cells infected with the *ssaV* strain (Fig. 8). SIF were detected in cells infected with the *aroA* strain, and the tubular aggregates were morphologically indistinguishable from SIFs observed in WT-infected HeLa cells. In contrast, no SIF formation was observed in cells infected with the *purD* strain. The detailed inspection of the membrane organization around intracellular *S.* Typhimurium indicated that the majority of the bacteria were enclosed by LAMP-2-positive membranes, indicating that all strains investigated here are capable in maintaining the SCV in infected HeLa cells (Fig. 8A).

Taken together, these analyses indicated that deficiency of *aroA* does not affect SPI2 function of intracellular *S.* Typhimurium in HeLa cells or RAW macrophages. The *purD*-deficient strain showed reduced SPI2-T3SS functions in HeLa cells and strong attenuation in RAW cells.

## Discussion

Our systematic analyses of the interaction of *S.* Typhimurium with various cell line cells indicated that the ability to invade host cells and to survive and proliferate intracellular is highly dependent on the type of host cells. The SPI1-T3SS-mediated invasion of epithelial cells is highly increased if polarized epithelial cells models are applied. The absolute levels of internalization were up to 50-fold higher for polarized cells compared to the commonly used non-polarized cell line HeLa. These observations suggest that *S.* Typhimurium is highly adapted to enter polarized cells from the apical side. This form of invasion cannot be sufficiently simulated in non-polarized cell models, resulting in much lower levels of internalization. The apical brush border and tight cell-to-cell contacts formed by polarized cell monolayers probably act as barriers against invading pathogens [23], yet the invasion of these cells by *S.* Typhimurium appears to be very efficient. However, the underlying molecular mechanisms resulting in the different efficiency of invasion are not known so far. We have recently identified SiiE as an adhesin specifically required for the interaction of *S.* Typhimurium with the apical side of polarized cells [8]. This observation indicates that distinct subsets of virulence factors are required for interaction with polarized and non-polarized cells.

After internalization, the intracellular fate of *S.* Typhimurium is also distinct in polarized and non-polarized epithelial cells. *S.* Typhimurium rapidly initiates intracellular replication in the non-polarized cell line HeLa, and this replication is dependent on the function of the SPI2-T3SS. However, intracellular proliferation is far less pronounced in the various polarized epithelial cell lines investigated here. Most interestingly, the proliferation of *S.* Typhimurium in polarized epithelial cells is independent from the function of the SPI2-T3SS. However, the T3SS-dependent translocation of SPI2 effector proteins was observed in infected polarized cells (SUH and MH, unpublished observations). It is not known if SPI2-dependent phenotypes, for example induction of SIF, juxtanuclear positioning of the SCV, or reorganization of the microtubule and actin cytoskeleton (reviewed in [3], [4]) also occur in polarized epithelial cells. Intracellular replication in polarized epithelial cells is only slightly affected by auxotrophies, while the *purD* mutation strongly reduced intracellular proliferation in HeLa cells. *purD* was previously identified as required for survival in macrophages [20]. This phenotype was explained by the reduced ability to synthesize mRNA and in turn proteins under intracellular conditions. This also resulted in the absence of a large number of stress proteins [20]. In line with these findings, we observed reduced SPI2-T3SS-dependent effector translocation and no induction of SIF by intracellular *purD*-deficient *S.* Typhimurium. Thus, one explanation of the intracellular defect of the *purD* strain might be the deficiency to expression genes of the SsrAB virulon and to synthesize the SPI2-T3SS and cognate effector proteins. However, the *purD* strain was killed in RAW macrophages while this strain survived and showed residual replication in epithelial cells. The intracellular virulence attenuation of the *purD* strain in macrophages was independent of the growth phase of infecting *S.* Typhimurium. These results indicate that the intracellular supply of nucleotide precursors is distinct in macrophages and epithelial cells.

We also observed a remarkable difference in the requirement for *aroA*-encoded 3-phosphoshikimate 1-carboxyvinyltransferase in epithelial cells and macrophages. The *aroA* mutation reduced intracellular replication in macrophages to level of the *ssaV* mutants or below, while replication was higher than of the WT strain in HeLa cells. *aroA* function is required for the synthesis of aromatic amino acids, as well as for the biosynthesis of pABA and DHBA as precursors of folate and enterochelin biosynthesis, respectively [6], [24]. The addition of pABA and/or DHBA to cell culture medium did not restore cultural growth to WT levels, indicating that limitation for aromatic amino acids may be the more relevant limitation for intracellular growth. Such putative amino acid limitation, however, would apply to intracellular *S.* Typhimurium within RAW macrophages, but not to those within HeLa cells. These findings may indicate differences in the supply of amino acids to the SCV in RAW and HeLa cells. In addition, the higher endocytic turnover in macrophages compared to epithelial cells has to be considered. Sebkova et al. [25] reported that *aro* mutations also cause outer membrane and cell wall defects in *S.* Typhimurium. These defects may also explain the higher susceptibility to antimicrobial factors in macrophages.

We and others previously observed the ability of *S.* Typhimurium to replicate in macrophages and the restriction of intracellular proliferation in murine dendritic cells [21], [26]. Growth restriction was also observed in fibroblasts, and specific mutations result in the release of this arrest and the overgrowth of intracellular *S.* Typhimurium in fibroblasts [27]. Based on the data reported here, we would extend the growth restriction phenotype also to *S.* Typhimurium in polarized epithelial cells. The ability of an intracellular pathogen to adjust and to restrict intracellular replication is considered as an important factor for successful pathogenesis (for further discussion of this issue, see [9], [28], [29]).

The results of this study suggest that the interpretation of infection experiments has to consider the specific feature of the cell line used. Recent studies also demonstrated that the growth kinetics of *S.* Typhimurium in infected host tissue is highly divergent from the massive replication observed in most cell culture models [10], [30]. Many aspects of the cellular microbiology and pathogenesis of *S. enterica* and other bacterial pathogens have been revealed by simple cells culture models such as infection of HeLa cells. Yet, the limitations of these models have to be considered and there is a clear demand for improved cell culture models for infection that provide physiological situations more close to those of an infected host tissue. The use of polarized epithelial cell cultures is one important step in this direction. Even more tissue-like conditions may be simulated by co-culture models resulting in induction of M-cells [31], [32], or use of polarized epithelial cells that form an apical mucus layer [33], [34]. Application of such advanced cell culture models should reveal more of the complexity of host-pathogen interplay.
